# Pre-transplant CRP–albumin ratio as a biomarker in patients receiving haploidentical allogeneic hematopoietic transplantation: Developing a novel DRCI-based nomogram

**DOI:** 10.3389/fimmu.2023.1128982

**Published:** 2023-02-15

**Authors:** Kejing Wang, Xing Jian, Ziwei Xu, Huafang Wang

**Affiliations:** ^1^Institute of Hematology, Union Hospital, Tongji Medical College, Huazhong University of Science and Technology, Wuhan, China; ^2^Collaborative Innovation Center of Hematology, Huazhong University of Science and Technology, Wuhan, China

**Keywords:** HSCT, inflammation, nutrition, disease risk comorbidity index, CAR

## Abstract

**Background:**

In allogeneic hematopoietic stem cell transplantation (allo-HSCT), prognostic indicators effectively predict survival. The Disease conditions prior to transplantation dramatically affects the outcome of HSCT. Optimization of the pre-transplant risk assessment is critical for enhancing allo-HSCT decision-making. Inflammation and nutritional status play significant roles in cancer genesis and progression. As a combined inflammatory and nutritional status biomarker, the C-reactive protein/albumin ratio (CAR) can accurately forecast the prognosis in various malignancies. This research sought to examine the predictive value of CAR and develop a novel nomogram by combining biomarkers and evaluating their importance following HSCT.

**Methods:**

Analyses were conducted retroactively on a cohort of 185 consecutive patients who underwent haploidentical hematopoietic stem cell transplantation (haplo-HSCT) at Wuhan Union Medical College Hospital during the period from February 2017 to January 2019. Of these patients, 129 were randomly assigned to the training cohort, and the remaining 56 patients constituted the internal validation cohort. Univariate and multivariate analyses were carried out to examine the predictive significance of clinicopathological factors in the training cohort. Subsequently, the survival nomogram model was developed and compared with the disease risk comorbidity index (DRCI) using the concordance index (C-index), calibration curve, receiver operating characteristics (ROC) curve, and decision curve analysis (DCA).

**Results:**

Patients were separated into low and high CAR groups using a cutoff of 0.087, which independently predicted overall survival (OS). Based on risk factors, CAR, the Disease Risk Index(DRI), and the Hematopoietic Cell Transplantation–specific Comorbidity Index(HCT-CI), the nomogram was developed to predict OS. The C-index and area under the ROC curve confirmed the improved predictive accuracy of the nomogram. The calibration curves revealed that the observed probabilities agreed well with those predicted by the nomogram in training, validation and entire cohort. It was confirmed by DCA that the nomogram offered greater net benefits than DRCI among all cohorts.

**Conclusion:**

CAR is an independent prognostic indicator for haplo-HSCT outcomes. Higher CAR was related to worse clinicopathologic characteristics and poorer prognoses in patients underwent haplo-HSCT. This research provided an accurate nomogram for predicting the OS of patients following haplo-HSCT, illustrating its potential clinical utility.

## 1 Introduction

One of the most promising treatments for patients with hematologic malignancies is allogeneic hematopoietic stem cell transplantation (allo-HSCT) (1). Pluripotent hematopoietic stem cells from human leukocyte antigen (HLA)-matched sibling donors have become critical options in transplant procedures (2). Owing to the shortage of HLA-matched sibling donors and unrelated donors, haploidentical HSCT (haplo-HSCT) protocols are being utilized globally. Anti-thymocyte globulin (ATG) and granulocyte colony-stimulating factor (G-CSF) were applied by Peking University researchers to develop a haplo-HSCT procedure and promote immune tolerance (3). The Beijing Protocol has played a vital role in haplo-HSCT in China.

It can be challenging to select patients who will benefit from HSCT because survival after transplantation can vary greatly and rely on many factors. Various factors, including age at HSCT, complications, the donor type, the disease status as well as the conditioning regimen, can affect outcomes (4, 5). Accumulating evidence suggests that inflammation has an essential role in cancer occurrence and progression (6). C-reactive protein (CRP) has been recognized for a long time as a marker of systemic inflammatory response, and comprehensive investigations have established its association with short survival rates in patients with various types of cancer (7–9). Yamamoto et al. found that the CRP level before transplantation was an independent prognostic factor for overall survival (10). There are a variety of factors that affect the nutritional condition of patients undergoing allogeneic HSCT, including intense chemotherapy, persistent nausea, severe infections, and the donor search time. Additionally, numerous studies have indicated that the pre-operative nutritional status, including albumin levels (11, 12), weight loss (13), and low body mass index (BMI), is related to poor clinical prognosis for patients following HSCT. Chee et al. confirmed that albumin < 30 g/L is the significant predictor of OS before HSCT (14). Recently, Multiple cancer studies have demonstrated that the pre-transplant CRP/albumin ratio (CAR), as a composite index of statistical inflammation and nutritional condition, is an independent predictive predictor (15–17).

To provide a score for pre-transplant risk and to better predict outcomes after allo-HSCT, several prognostic models have been performed. The Hematopoietic Cell Transplantation–specific Comorbidity Index (HCT-CI) and its derivation, the Comorbidity–Age Index, are derived from the complication condition of the patient (18). The Disease Risk Index (DRI) effectively stratifies the outcome of haplo-HSCT plus post-transplant cyclophosphamide, as reported by McCurdy et al (19). The most prevalent prognostic scores in forecasting clinical results after allo-HSCT is the European Group for Blood and Marrow Transplantation (EBMT) risk score, derived from an investigation of chronic myeloid leukemia transplant recipients, which could forecast survival and mortality for a range of patients with hematologic malignancies. Wang et al. (20) proposed the haplo-EBMT score based on the EBMT score. Recently, Bejanyan et al. (21) developed an entirely new predictive measure termed the disease risk comorbidity index (DRCI), which incorporates both DRI and HCT-CI, and tested its prognostic accuracy in patients receiving peripheral blood or bone marrow from ISD, URD, or umbilical cord blood. This model was subsequently validated in a haplo-HSCT cohort (22). However, these models do not consider the pre-transplant inflammatory and nutritional status.

A thorough pre-transplant evaluation is essential at the time of assessing the risks and advantages of haplo-HCT. Therefore, It would be of tremendous clinical benefit for patients undergoing haplo-HSCT to develop a well-rounded pre-HSCT prognostic system that considers the risk elements associated with the patient and the disease. According to our knowledge, the prognostic implications of CAR in patients who received haplo-HSCT is unknown. Therefore, we hypothesized that integration of the inflammatory state, malnutrition, disease characteristics and comorbidities could provide a more comprehensive and precise prediction of overall survival (OS) than DRCI.

## 2 Materials and methods

### 2.1 Study patients

**Figure 1:**
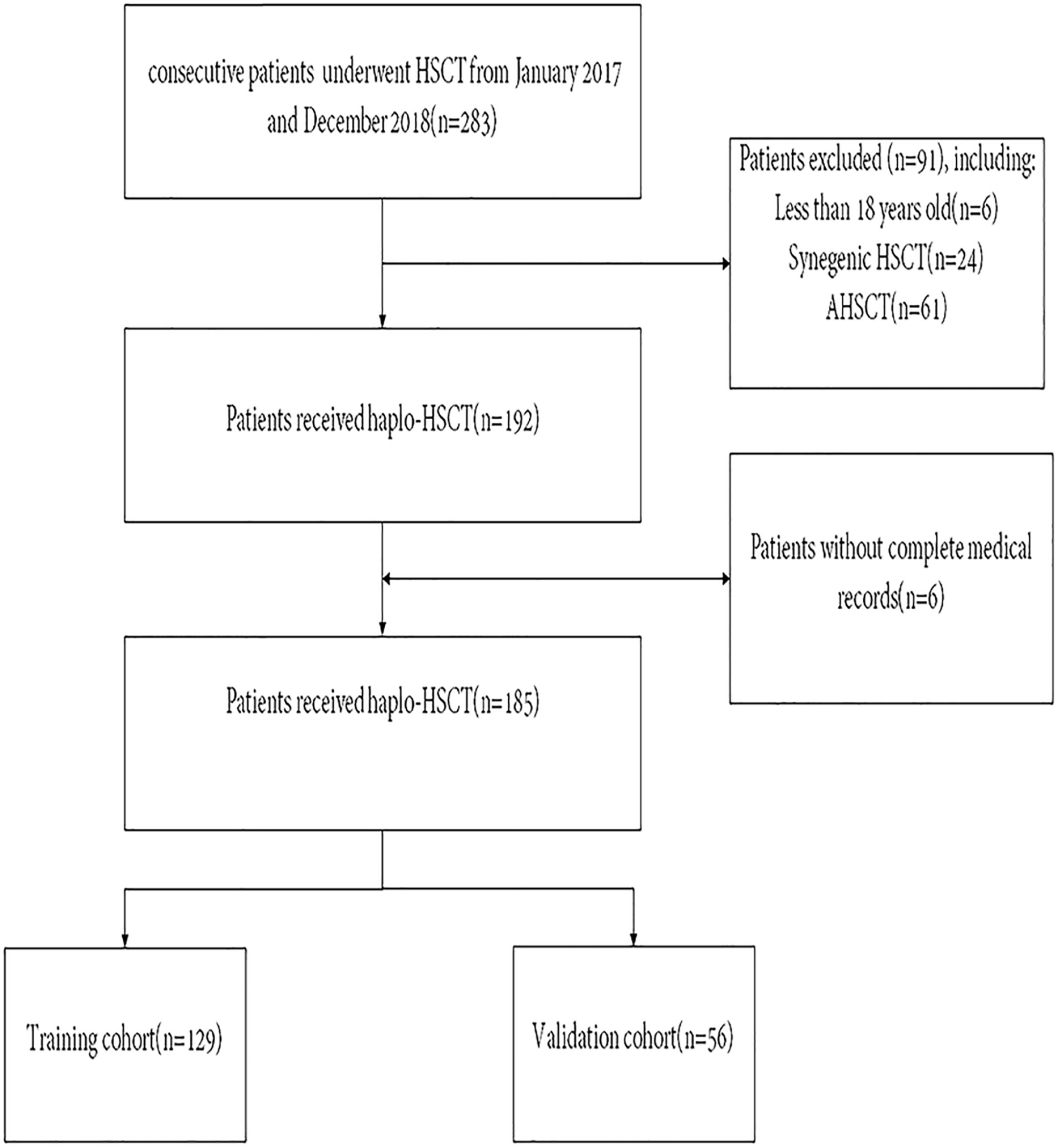
Patient flow diagram. HSCT, Hematopoietic Stem Cell Transplantation. AHSCT, Autologous Hematopoietic Stem Cell Transplantation.

This research comprised all consecutive adult patients (aged >18 years) with malignant hematologic disease who underwent haplo-HSCT within the period of February 2017 to January 2019 at Wuhan Union Medical College Hospital. All patients who received haplo-HSCT as their initial allogeneic transplant were included in the risk assessment score and outcome analysis. Patients who received a second or third HSCT and those with incomplete medical records were omitted from this work. The flow diagram of the patient is illustrated in **Figure 1**. Clinical data are acquired retrospectively by consulting the medical documentation of each patient. Our last follow-up for the endpoint analysis was performed on January 31, 2022. This research was authorized by the Ethics Committee of Tongji Medical College, Huazhong University of Science and Technology and was conducted following the principles of the Declaration of Helsinki.

### 2.2 Definitions

All data were collected directly within 10 days of the allo-HSCT conditioning regimen to assess the inflammatory and nutritional status before transplantation. We investigated CRP as a marker of inflammation and albumin as an indicator of nutritional status. CAR was counted by dividing the serum CRP level by the serum albumin level as a combination marker. DRI was applied by the criteria according to Armand et al (23). HCT-CI was used to evaluate the comorbidities of HSCT recipients (24). OS was computed from the date of haplo-HSCT to that of death or the last date the patient was known to be alive.

### 2.3 CRP and albumin level measurement

5 ml of cubital vein whole blood was extracted from all patients in the morning on an empty stomach. Serum CRP level measurements were included in the routine clinical tests using an automated biochemical analyzer *via* immunoturbidimetric assay. Serum albumin was measured by means of the bromocresol purple method using fully automated equipment (all kits were purchased from Roche Diagnostics).

### 2.4 Donor selection, transplant regimens, and GVHD prophylaxis and treatment

Donor selection method in accordance with the non-HLA system (25). The Standards proposed by International Center for Blood and Marrow Transplantation were utilized to classify the intensity of the conditioning regimens (26). Most of the patients adopted myeloablative conditioning regimen (MAC) comprising cytarabine (4 g/m2/day on days −10 to −9), busulfan (3.2 mg/kg/day, administered intravenously on days −8 to −6), cyclophosphamide (1.8 g/m2/day on days −5 to −4), and semustine (250 g/m2 on day −3), along with rabbit ATG (2.5 mg/kg/day from on days−5 to −2, thymoglobulin; Imtix Sangstat, Lyon, France) (27). The reduced intensive conditioning (RIC) comprised fludarabine (30 g/m2/day administered intravenously on days −10 to −5), busulfan (3.2 mg/kg/day administered intravenously on days −6 to −5), and ATG (5 mg/kg/day, administered intravenously on days −4 to −1). At 45–60 days following allo-HSCT, individuals with relapsed/refractory leukemia without GVHD or severe infection could undergo prophylactic G-CSF–stimulated donor leukocyte infusion (3). G-CSF was employed to stimulate myeloid and blood cells for the grafts.

### 2.5 Development of a prognostic model

In total, 185 patients were randomized into two groups (training and validation cohorts) based on a ratio of 7 to 3. the following medical characteristics were evaluated to identify the predictive factors of survival in the training cohort: age at HSCT, sex, DRI, HCT-CI, CAR, donor–recipient relationship, donor–recipient sex match, donor–recipient blood type match, number of HLA mismatches and conditioning regimens. The construction of nomograms is based on multivariate regression models (such as Cox and logistic regression models), which can may simplify and visualize complex regression equations, and making the results of prediction model more readable and valuable for use.Variables significant at P < 0.10 in univariate analysis as underlying survival risk elements were selected for multivariate Cox proportional risk regression to Identify independent prognostic factors. According to the above results, Statistically significant parameters (P<0.05) were incorporated into a nomogram to predict OS in patients after haplo-HSCT.

### 2.6 Model validation and clinical use

The predictive capability of the nomogram was evaluated by assessing discrimination power and calibration in the training, validation, and entire cohorts. The concordance index (C-index) was computed to evaluate the discriminative ability of the novel nomogram in all cohorts. A higher C-index suggests superior capability to differentiate patients according to survival status. Calibration curve produced from 1000 bootstrap replicates was used to examine the conformity between the model-predicted probability and the actual condition. In addition, to further evaluate the predictive performance of the nomogram for OS, ROC curve was plotted over the total score of the nomogram for OS for each patient, and AUC analyses was computed. Finally, the net benefit of the novel nomogram was measured with decision curve analysis (DCA).

### 2.7 Statistical analysis

Categorical features were presented as counts and proportions and examined by the chi-squared test. Continuously parameterized characteristics were reported as the median and interquartile range (IQR), and comparisons of continuous variables between cohorts were determined using the Mann–Whitney U test. In these tests, a two-tail P value of 0.05 was deemed to be statistically significant. Based on the maximum Youden index, the best cutoff value for continuous variables was determined by ROC curve analysis. OS rates were estimated using the Kaplan–Meier method, and Disparities between groups were evaluated by log-rank test. The prognostic index was calculated *via* Cox regression. The nomogram was plotted based on risk features of multivariate analysis with the rms26 package in R. The C-index was computed To test the model’s capabilities of Discrimination using the survival package in R. The rms package in R was utilized to formulate calibration plots. DCA curve was conducted in R with ggDCA package. R was employed for statistical studies (Version 4.1.2, R Foundation for Statistical Computing, Vienna, Austria).

## 3 Results

### 3.1 Optimal cutoff of CAR for predicting all-cause mortality following haplo-HSCT

**Figure 2:**
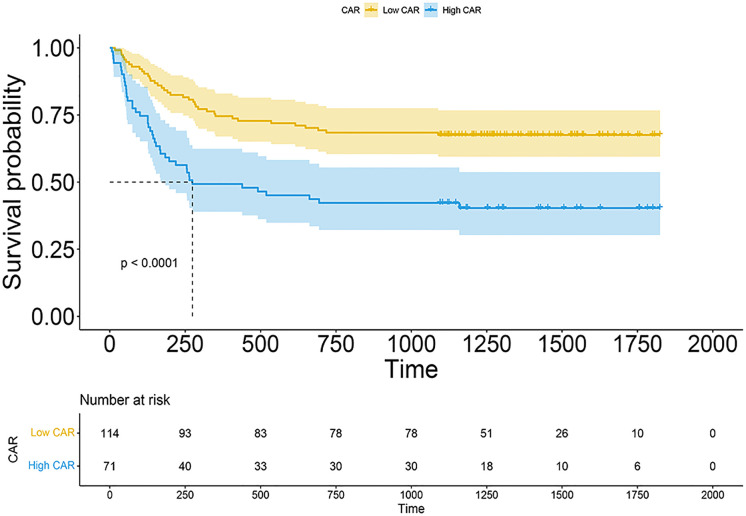
Kaplan–Meier plots of OS according to patient groups with low CAR or high CAR.

The median CAR among 185 patients was 0.087 (range, 0.00667–2.67352). With all-cause patient death as the outcome variable, a 3-year time-dependent ROC curve was created, and the maximum Youden index for OS was utilized to determine the optimal cutoff. The optimal cutoff of CAR was finally regarded as 0.109. Patients were then separated into low CAR and high CAR groups based on the appropriate cutoff for CAR. Kaplan–Meier survival curves demonstrated that patients in the high CAR group had a higher mortality rate (P < 0.0001, **Figure 2**). There were no statistically significant differences in age (P = 0.629), sex (P = 0.7591), and HCT-CI (P = 0.3313) between the high and low CAR groups. Conversely, DRI and underlying diseases significantly differed between the groups (both P < 0.05, **Table 1**).

**Table 1:** The relationship between CAR and clinical characteristics.

Variable	l	Low CAR group (n=114)	High CAR group (n=71)	P-value
age(years), median (IQR)		32.000 [26.000,46.00]	31.000 [25.000,44.500]	0.629
sex,n (%)	Male	0 (61.40)	46 (64.79)	0.7591
	female	44 (38.60)	25 (35.21)	
Underlying disease, n (%)	Acute leukaemia	33 (28.95)	31 (43.66)	0.0275
	Myelodysplastic syndrome	69 (60.53)	27 (38.03)	
	Myeloproliferative neoplasms	2 (1.75)	3 (4.23)	
	Non-Hodgkin lymphoma	10 (8.77)	10 (14.08)	
HCT-CI scores,n (%)	0(low risk)	59 (51.75)	29 (40.85)	0.3313
	1-2(intermediate risk)	52 (45.61)	39 (54.93)	
	>3(high risk)	3 (2.63)	3 (4.23)	
DRI,n (%)	Low risk	2 (1.75)	1 (1.41)	0.0015
	Intermediate risk	91 (79.82)	41 (57.75)	
	High risk	19 (16.67)	19 (26.76)	
	Very high risk	2 (1.75)	10 (14.08)	

### 3.2 Baseline clinical features of the research cohort

The baseline clinical features of the training and validation cohorts are depicted in **Table 2**. Following haplo-HSCT, 44 patients relapsed, and 35 died of non-relapse related diseases. Meanwhile, 59 patients were enrolled in the validation cohort. The median follow-up times were 1386 days (range, 7–1825) for the training cohort and 1421 days (range, 38–1825) for the validation cohort. The OS rates at three years after haplo-HSCT were 58.9% [95% confidence interval (CI) = 54.6%–63.2%] in the training cohort and 55.4% (95% CI = 0.488%–62%) in the validation cohort. Clinical baseline information did not differ between the cohorts (**Table 2**).

**Table 2:** Patients’ baseline features.

Variable		Training cohort(n=129)	Validation cohort(n=56)	P-value
age (years),(median [IQR])		32.000 [25.000, 46.000]	30.000 [26.000, 42.250]	0.722
sex,n (%)	Male	80 (62.02)	36 (64.29)	0.898
	female	49 (37.98)	20 (35.71)	
Underlying disease,n (%)	Acute leukaemia	42 (32.56)	22 (39.29)	0.624
	Myelodysplastic syndrome	67 (51.94)	29 (51.79)	
	Myeloproliferative neoplasms	4 (3.10)	1 (1.79)	
	Non-Hodgkin lymphoma	16 (12.40)	4 (7.14)	
HCT-CI scores,n (%)	0(low risk)	62 (48.06)	26 (46.43)	
	1-2(intermediate risk)	62 (48.06)	29 (51.79)	
	>3(high risk)	5 (3.88)	1 (1.79)	0.720
DRI,n (%)	Low risk	3 (2.33)	0 (0.00)	0.284
	Intermediate risk	92 (71.32)	40 (71.43)	
	High risk	28 (21.71)	10 (17.86)	
	Very high risk	6 (4.65)	6 (10.71)	
CAR, (median [IQR])		0.088 [0.076, 0.175]	0.085 [0.075, 0.222]	0.751
CAR status, n (%)		81 (62.79)	33 (58.93)	0.740
		48 (37.21)	23 (41.07)	
Donor-recipient relationship,n (%)	Father-child	26 (20.16)	12 (21.43)	0.4803
	Mother-child	6 (4.65)	4 (7.14)	
	Sibling–sibling	47 (36.43)	25 (44.64)	
	Child–parent	47 (36.43)	13 (23.21)	
	Collateral related donor	3 (2.33)	2 (3.57)	
Donor-recipient sex-matched,n (%)	Male-male	52 (40.31)	25 (44.64)	0.695
	Male–female	35 (27.13)	11 (19.64)	
	Female–male	28 (21.71)	12 (21.43)	
	Female-female	14 (10.85)	8 (14.29)	
Number of HLA-A, HLA-B, HLA-DR mismatches,n (%)	0-2	51 (39.53)	24 (42.86)	0.795
	3	78 (60.47)	32 (57.14)	
Donor-recipient blood type matched,n (%)	Matched	69 (53.49)	32 (57.14)	0.866
	Major mismatched	17 (13.18)	5 (8.93)	
	Minor mismatched	26 (20.16)	11 (19.64)	
	Major–minor mismatched	17 (13.18)	8 (14.29)	
Conditioning regimen,n (%)	Chemotherapy based regimen	103 (79.84)	39 (69.64)	0.186
	TBI based regimen	26 (20.16)	17 (30.36)	

Minor ABO mismatch demonstrated the donor had isohemagglutinins against the recipient’s red blood cells, including the following blood group combinations: O (donor) into A, B, or AB (recipient); A or B (donor) into AB (recipient) (recipient). Major ABO mismatched indicated that the recipient had isohemagglutinin resistant to donor red blood cells and included the following blood group combinations: A, B, or AB (donor) became O (recipient), and AB (donor) became A or B (recipient). Major-minor mismatched suggest that both the donor and the recipient share isohemagglutinins: A to B and vice versa.

### 3.3 Construction of the novel nomogram of OS

**Figure 3:**
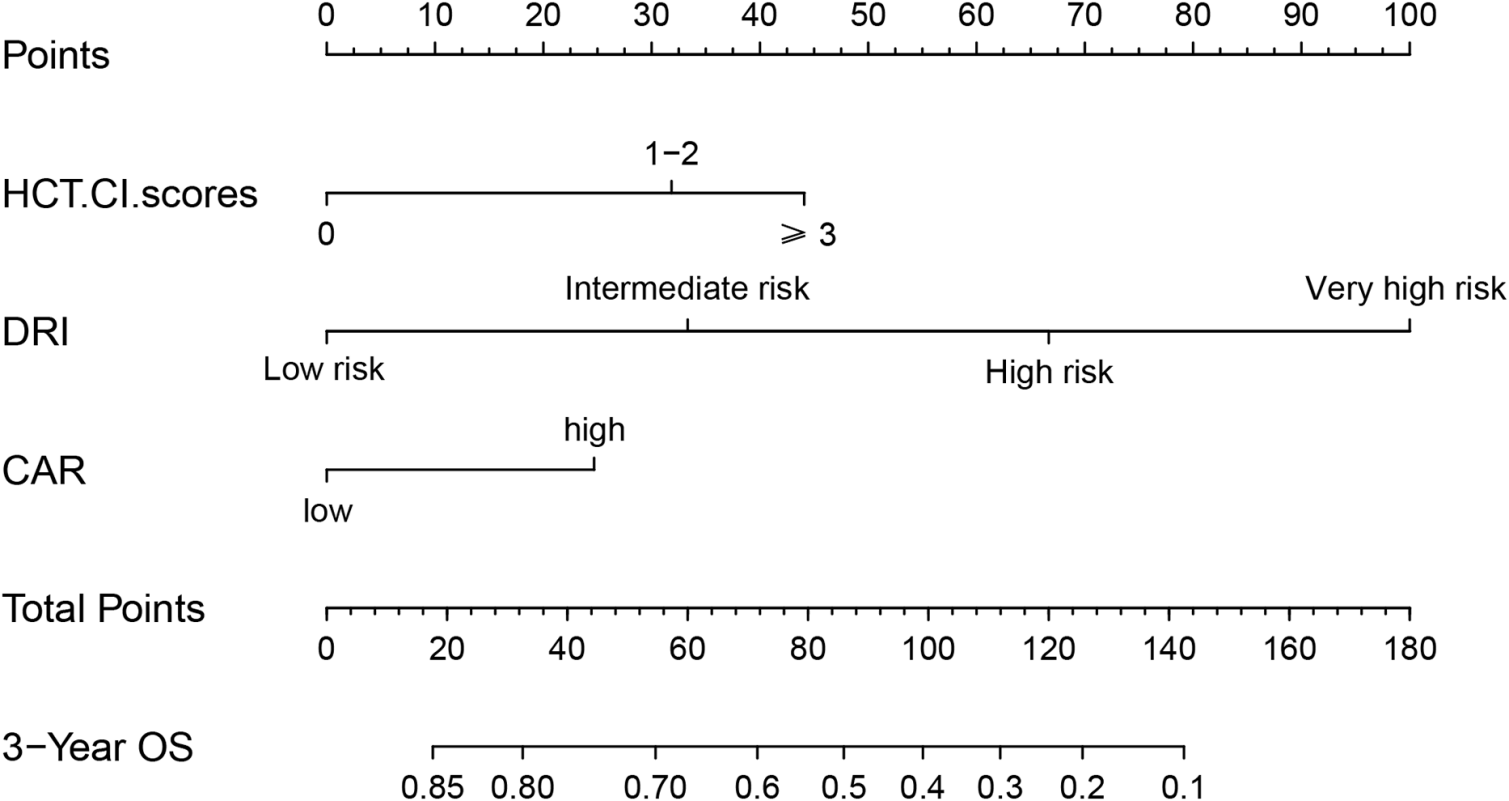
Nomogram of OS for patients with haplo-HSCT. Low CAR: ≤0.109; High CAR: >0.109.

In univariate analysis, OS was related to DRI (P < 0.001), HCT-CI (P = 0.038), and CAR (P < 0.001). In multivariate Cox regression analysis, DRI (P < 0.001), HCT-CI (P = 0.011), and CAR (P =0.017) were independent predictors of OS following haplo-HSCT (**Table 3**). These independent variables were incorporated into the OS rate estimation nomogram (**Figure 3**). For each patient, the scores of the three risk factors indicated by the scale were added together. Then, OS was calculated using the nomogram’s “total score” axis.

**Table 3:** Univariate and multivariate Cox analysis for OS in patients with haplo-HSCT in training cohort.

Variable	Univariate		Multivariate	
	HR (95% CI)	p-value	HR (95% CI)	p-value
HCT-CI scores	1.283(1.014-1.624)	0.038	1.366(1.074-1.736)	0.011
DRI	2.273(1.713-3.017)	0	2.057(1.525-2.776)	0
CAR status	0.411(0.264-0.641)	0	0.562(0.350-0.903)	0.017
age	1.012(0.993-1.032)	0.227		
sex	0.756(1.014-1.624)	0.243		
Donor–recipient relationship	1.126(0.921-1.376)	0.248		
Donor–recipient sex-matched	0.904(0.731-1.118)	0.352		
Number of HLA-A, HLA-B, and HLA-DR mismatches	1.210(0.817-1.793)	0.342		
Donor–recipient blood type matched	0.607(0.867-1.278)	0.607		
HLA mismatches	1.033(0.841-1.268)	0.759		
Conditioning regimen	0.744(0.452-1.227)	0.247		

### 3.4 Validation of the novel nomogram

**Figure 4:**
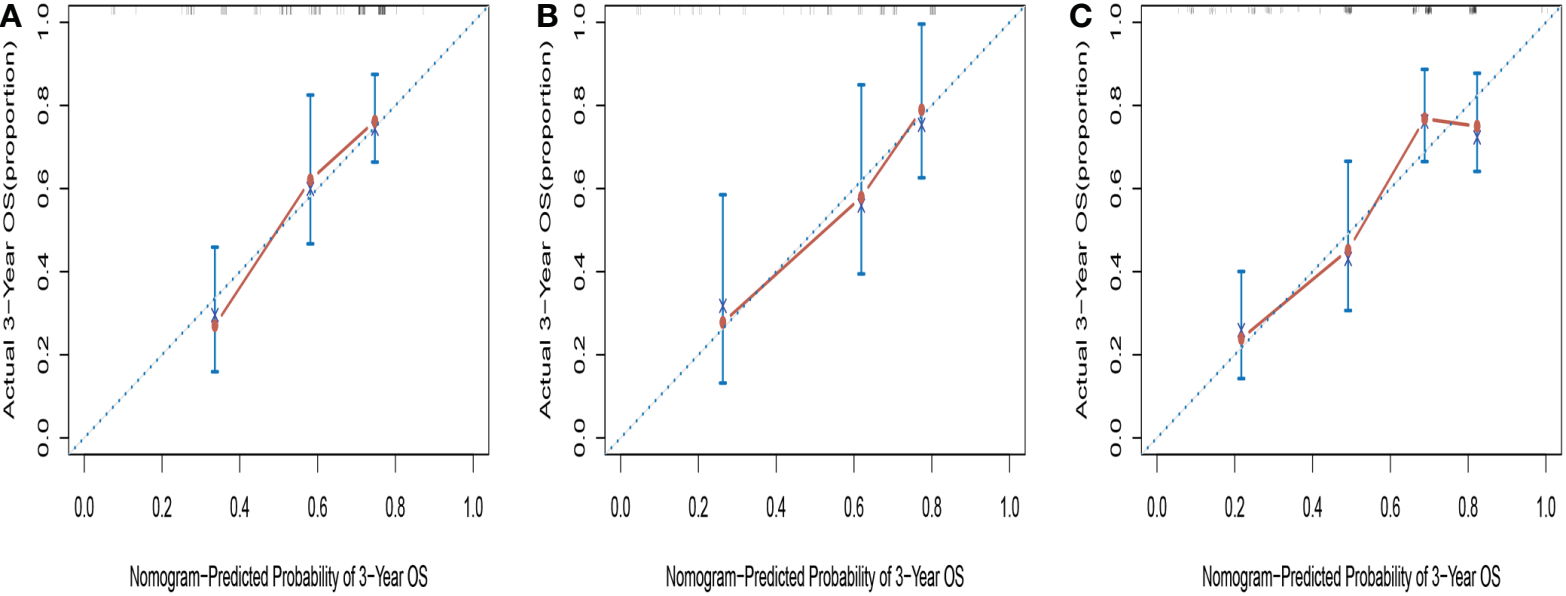
Calibration curves for predicting 3-year OS in training cohort **(A)**, validation cohort **(B) **and entire cohort **(C)**.

The C-indices of the novel nomogram for 3-year OS in the training cohort by bootstrap resampling was 0.671 (95% CI = 0.5923–0.7496). Meanwhile, it was found that the calibration plot displayed good similarity between the predicted and actual survival rates (**Figure 4A**), which indicated that the spots were close to the 45-degree line. The C-indices of the nomogram for the validation and entire cohorts were 0.7007 (95% CI: 0.5858–0.8156) and 0.7058 (95% CI:0.6465–0.7651), respectively. Additionally, the calibration plots for the validation (**Figure 4B**) and entire cohorts (**Figure 4C**) indicated good consistency between the anticipated and actual observations.

### 3.5 Comparison of the nomograms and DRCI

**Figure 5:**
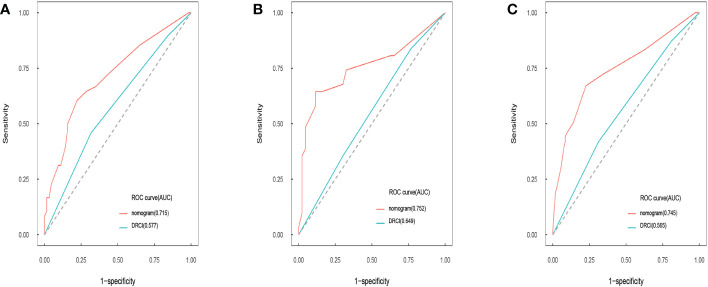
Area under the ROC curves of nomogram and DRCI of OS in training cohort **(A)**, validation cohort **(B)**, and entire cohort **(C)**.

**Figure 6:**
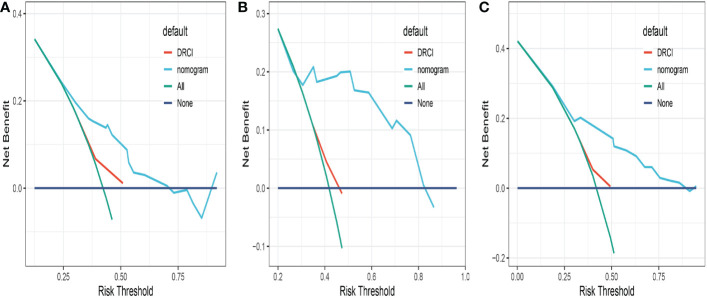
Comparison of the decision curves between the nomogram and the DRCI in the training **(A)**, validation **(B)** and entire cohort **(C)**.

Comparisons between DRCI and the nomograms were also conducted. For the three cohorts shown in the figure, the AUCs of the nomogram were greater than those of DRCI [0.715 (95% CI:0.617–0.812) *vs*. 0.577 (95% CI:0.479-0.676); 0.752 (95% CI:0.622–0.884) *vs*. 0.649 (95% CI:0.521–0.777); and 0.745 (95% CI:0.672–0.818) *vs*. 0.565 (95% CI:0.488–0.641), respectively], indicating that the nomogram had better discriminative potential(**Figures 5A-C**). The 3-year DCA illustrated that the prognostic model had greater net benefit than DRCI within a wide range of tolerable threshold probabilities in the training, validation, and entire cohorts(**Figures 6A-C**). These findings adequately demonstrate that the innovative prognostic model predicted OS more accurately than DRCI (**Figure 5**).

## 4 Discussion

DRI, HCT-CI, and CAR were the components of the developed OS nomogram. Previous studies using multivariate analysis also demonstrated significant associations between these prognostic variables and OS in patients following haplo-HSCT. Our results reconfirmed the conclusions of these investigations. In this research, we found that CAR was negatively correlated with OS following haplo-HSCT. Furthermore, we proposed a nomogram for stratifying patients at the time of haplo-HSCT by incorporating CAR into the established DRCI scoring system. The nomogram outperformed the DRCI in predicting OS in haplo-HSCT cohorts, which gave clinicians more flexibility in making personalized decisions about the medical management of patients after haploidentical HSCT.

DRI, which originated from the underlying hematological malignancy, cytogenetics for acute myeloid leukemia and myelodysplastic syndromes, and disease status at the time of transplantation, is a validated tool commonly used to predict survival outcomes following HSCT. This score is available for patients receiving HSCT independently of age, the pretreatment intensity regimen, and the graft source (22, 23). Several researchers verified their retrospective series using the original DRI (28) or refined DRI (23) and demonstrated its prognostic validity for several types of HSCT, including T-cell–depleted (29), haploidentical, and umbilical cord blood transplantation (30).

The HCT-CI is a modified version of the Charlson Comorbidity Index (24), which is calculated by considering 17 pre-transplant complications affecting 2-year NRM in a group of 1055 patients who received allo-HSCT (24). Several studies evaluated the predictive value of HCT-CI and discovered that patients with high-risk diseases show a noticeably worse prognosis than those with standard-risk diseases (18, 23). Several investigations have documented that certain comorbid conditions, including chronic lung disease, diabetes and obesity, may be involved in the development and progress of hematological malignancies. Sorror et al. (31) also noted that in addition to having the lowest survival rates, patients with severe comorbidities and disease burdens appear to have a decreased risk of non-relapse mortality after non-myeloablative treatment in comparison to conditioning.

According to studies, malnutrition and systemic inflammation are significantly associated with worse outcomes in patients who underwent HSCT (32, 33). Reduced albumin levels are correlated with malnutrition and systemic inflammation (6, 33). Furthermore, elevated CRP levels are linked with lymphopenia and impaired T-cell responses in tumors, which might further promote cancer progression (34). Instead of analyzing each element separately, CRP and albumin levels were analyzed together. It has been found that CAR can forecast the outcome of patients received HSCT with solid cancer (34) and hematologic malignancies (35). Its prognostic value was recently validated in elderly patients with acute myeloid leukemia who did not undergo HSCT (36). Consistent with our conclusion, higher CAR, indicating hypoalbuminemia and elevated CRP levels, is related to worse survival in patients following HSCT.

The cutoff of CAR has differed among studies (37–39). Variations in cutoffs can be attributed to differences in patient groups, diagnostic methods, and clinical practices. Because the majority of pertinent publications are single-center studies, there is currently no consensus conclusion on whether these cutoffs apply to the prognosis of all patients with HSCT. In other investigations, patients in the high CAR group experienced more aggressive tumor behavior (38)and a shorter survival time (37, 39). Determining the ideal cutoff of CAR is crucial for its clinical application, and this research might offer a better option for identifying the optimal cutoff of CAR.

The precise mechanism underlying the role of CAR in cancer progression is uncertain and potentially complex. Our investigation demonstrated that high CAR was significantly related to DRI. (P = 0.0015), suggesting that higher CAR is correlated with more invasive cancer behavior in patients following haplo-HSCT. This might be explained by the possibility that tumor cells release cytokines that induce CRP elevation or that CRP elevation is a substituted marker for the active release of cytokines that promote tumor cell proliferation (40, 41).

There are some limitations to this study, despite our nomogram incorporating CAR having significantly better predictive accuracy than DRCI. First, This research is retrospective rather than prospective, which might have resulted in data bias. Furthermore, the study relied on clinical data from our single center database, and there was no external validation. Third, during the duration of the study, nutritional interferences, for example, using intravenous supplementation for patients with decreased food intake, was an integral element of the standard treatment. However, In this study, it was not possible to analyze the influences of these interferences on inflammatory and nutritional conditions and transplantation outcomes. Thus, the results of our research require additional confirmation *via* prospective research at other institutions, and validation of the nomogram requires larger quantities of patients and more long-term follow-up to obtain more persuasive clinical results. Finally, several molecular markers might predict patient relapse and survival, and it may be helpful to further stratify the patients.

## 5 Conclusion

To our knowledge, none of the existing scoring systems considers the pre-transplant inflammatory and nutritional status. This is the first study to demonstrate the predictive power of pre-transplant CAR for survival outcomes following haplo-HSCT. Compared with DRCI, the nomogram incorporating CAR displayed substantially improved predictive accuracy. It has been proven that a high CAR at diagnosis is associated with a poor prognosis following haplo-HSCT, and this important finding can lead to further investigation.

## Data availability statement

The original contributions presented in the study are included in the article/supplementary material. Further inquiries can be directed to the corresponding author.

## Ethics statement

The studies involving human participants were reviewed and approved by the Ethics Committee of Tongji Medical College of Huazhong University. The patients/participants provided their written informed consent to participate in this study.

## Author contributions

KW collected, analyzed the data, and wrote the paper. XJ and ZX researched the literature and revised the paper. All authors contributed to the article and approved the submitted version.
