# Corrigendum: Pre-transplant CRP–albumin ratio as a biomarker in patients receiving haploidentical allogeneic hematopoietic transplantation: developing a novel DRCI-based nomogram

**DOI:** 10.3389/fimmu.2023.1206392

**Published:** 2023-05-02

**Authors:** Kejing Wang, Xing Jian, Ziwei Xu, Huafang Wang

**Affiliations:** ^1^Institute of Hematology, Union Hospital, Tongji Medical College, Huazhong University of Science and Technology, Wuhan, China; ^2^Collaborative Innovation Center of Hematology, Huazhong University of Science and Technology, Wuhan, China

**Keywords:** HSCT, inflammation, nutrition, disease risk comorbidity index, CAR

In the published article, there was an error in affiliation 1. Instead of “Institute of Hematology, Wuhan Union Hospital, Tongji Medical College, Huazhong University of Science and Technology, Wuhan, China,”, it should be “Institute of Hematology, Union Hospital, Tongji Medical College, Huazhong University of Science and Technology, Wuhan, China”.

The authors apologize for this error apologize for this error and state that this does not change the scientific conclusions of the article in any way. The original article has been updated.

